# Successful Boutonniere Reconstruction With Wide-Awake Local Anesthesia No Tourniquet

**DOI:** 10.1016/j.jhsg.2022.05.001

**Published:** 2022-05-27

**Authors:** Can Emre Bas, Egemen Ayhan, Orhan Kunu, Cigdem Ayhan Kuru

**Affiliations:** ∗ Department of Orthopedics and Traumatology, Hand Surgery, University of Health Sciences, Diskapi Yildirim Beyazit Training and Research Hospital, Ankara, Turkey; † Department of Physiotherapy and Rehabilitation, Faculty of Health Sciences, Hacettepe University, Ankara, Turkey

**Keywords:** Boutonniere deformity, Central slip, Relative motion orthosis, WALANT

## Abstract

**Purpose:**

In this retrospective study, we evaluated the results of central slip reconstruction with a modified Snow’s technique under wide-awake local anesthesia no tourniquet.

**Methods:**

Between 2016 and 2019, 13 patients with boutonniere deformity were operated. All of the patients had boutonniere deformity with a passively correctable proximal interphalangeal joint. In 7 patients, temporary proximal interphalangeal joint transfixation with a K-wire to secure the repair was preferred. For the rest of the patients, postoperative follow-up was done with an orthosis. All patients were referred to a hand therapist for postoperative rehabilitation.

**Results:**

The mean interval between the injury time and the surgery was 55.7 days. After the surgery, the average proximal interphalangeal joint flexion was 104.8° and the loss of proximal interphalangeal joint extension was 6.15°. The average distal interphalangeal flexion was 65.3°. Seven cases had excellent results, 4 patients had good results, and 2 patients had fair results.

**Conclusions:**

Our study demonstrated that with a modified Snow technique, encouraging results can be achieved for neglected central slip injuries in which primary repair is impossible. Surgery under wide-awake local anesthesia no tourniquet enables the surgeon to check the stability of the repair, and early active motion with relative motion flexion orthoses can be started with confidence.

**Type of study/level of evidence:**

Therapeutic IV.

Central slip (CS) injuries are easily overlooked in emergency services because the patient generally can extend their finger with intact lateral bands. In a few weeks, the lateral bands displace volarly and boutonniere deformity becomes evident.^1^ In these neglected injuries, any CS remnant for primary repair is hard to find. Thus, harvesting change can be made a tendinous structure is often required to treat chronic boutonniere deformities, and various results have been reported with different surgical techniques.^2^, ^3^, ^4^, ^5^, ^6^

Snow^7^ advised augmenting the primary repair of CS with a retrograde extensor tendon flap “as a batten” over the injury site. In his technique, no important tendinous structure was sacrificed, and augmented secure repair was obtained. This technique could also be applied for neglected CS injuries, with the further advantage of performing it under wide-awake local anesthesia no tourniquet (WALANT).

We hypothesized that the opportunity to evaluate the reconstructed CS through intraoperative active movement under WALANT would provide promising results in the treatment of boutonniere deformity. We aimed to present the outcomes after reconstruction of neglected CS injuries with a retrograde extensor tendon flap under WALANT.

## Materials and Methods

We operated on 13 patients with a boutonniere deformity between August 2016 and February 2019. All of the patients had a stage 2 boutonniere deformity with a passively correctable proximal interphalangeal (PIP) joint.^8^ The written informed consent of the patients was obtained, and the study was approved by the ethical committee of the University of Health Sciences, Diskapi Yildirim Beyazit Training and Research Hospital. All procedures followed were in accordance with the ethical standards of the responsible committee on human experimentation (institutional and national) and with the Helsinki Declaration of 1975, as revised in 2008. The inclusion criteria were as follows: having a boutonniere deformity with a passively correctable PIP joint, being >18 years, and being willing to be included in the study. The exclusion criteria were having accompanying injuries (flexor tendon, fracture, digital nerve, and digital artery injuries), skin loss requiring a graft or flap to cover the repair zone, and fixed PIP joint flexion contracture. The patients’ gender, age, injured finger, and injury mechanisms were documented in this retrospective study (Table 1).

**Table 1:** Patients’ Characteristics and Results

Patient	Sex	Age	Injury-Surgery Interval	Mechanism	Finger	Postoperative Immobilization Choice	Final Results			
							PIP Joint Extension Defect	PIP Joint Active Flexion	DIP Joint Active Flexion	Functional Score
1	M	71	30 d	Laceration	Index	Extension orthosis	0	105	70	Excellent
2	F	52	45 d	Laceration	Middle	Extension orthosis	0	110	70	Excellent
3	F	24	15 d	Laceration	Index	Extension orthosis	0	110	70	Excellent
4	M	36	90 d	Laceration	Middle	Transfixation	0	110	70	Excellent
5	M	36	20 d	Closed injury	Little	Transfixation	15	100	65	Fair
6	M	37	30 d	Laceration	Middle	Transfixation	10	100	60	Good
7	M	47	30 d	Laceration	Index	Transfixation	20	105	70	Good
8	M	51	45 d	Closed injury	Middle	Transfixation	0	103	75	Excellent
9	F	58	120 d	Laceration	Index	Transfixation	10	100	50	Good
10	F	39	180 d	Laceration	Index	Transfixation	20	95	60	Fair
11	F	20	60 d	Laceration	Index	RMF orthosis	0	115	70	Excellent
12	F	38	30 d	Laceration	Index	RMF orthosis	0	110	70	Good
13	M	40	30 d	Laceration	Index	RMF orthosis	5	100	50	Good

d, days.

The decision for either K-wiring or orthosis fabrication was based on compliance to postoperative hand therapy. Those with a low likelihood of attending postoperative hand therapy were K-wired, especially those with transport issues. Our clinic accepts many patients from other cities, and those patients may have interruptions during follow-up. We obliged to use K-wire fixation for such patients.

### Surgical technique

All of the surgeries were done under WALANT. The local anesthesia solution contained 1% lidocaine, 1:100,000 epinephrine, and 8.4% sodium bicarbonate.^9^ A digital block was performed with 2 cc of local anesthetic at the palmar side of the proximal phalangeal crease. After the digital block anesthesia, 4 cc of tumescent local anesthesia was injected dorsally beginning from the metacarpophalangeal (MCP) joint and advancing through the proximal phalanx to the distal interphalangeal (DIP) joint. Thirty minutes after the first injection, a lazy-S incision starting from the basis of the proximal phalanx and extending through the PIP joint was made. After passing the subcutaneous tissue and cauterizing the dorsal crossing veins, the extensor apparatus was isolated and the fibrotic tissue over the PIP joint was debrided. In cases when the primary repair was not possible, the distally based extensor tendon flap was designed with a number 11 surgical blade, starting from the extensor tendon at the basis of the proximal phalanx. The retrograde tendon flap had an average length of 1 cm and a width of 0.4 cm. The flap was sutured on the distal edge of the CS remnant with 4-0 polydioxanone. The donor site extensor tendon defect was closed with 4-0 polydioxanone, as well. The tension of the repair and any gap formation was checked with intraoperative, active movement under WALANT. In 7 patients, we performed temporary PIP joint transfixation with a K-wire to secure the repair after checking the stability of the repair with intraoperative, active motion. K-wire fixation was done in a retrograde manner from the ulnar or radial side of the middle phalanx to the proximal phalanx. The position of the K-wire was checked under the fluoroscopy. For the rest of the patients, postoperative follow-up was done with either an orthosis that kept the PIP and MP joints at full extension (in 3 patients) or with relative motion flexion (RMF) orthoses (in 3 patients). The skin was closed with 4-0 nonabsorbable sutures. All patients were referred to a hand therapist (C.A.K.) for postoperative rehabilitation.

### Rehabilitation protocols

The main objectives of postoperative rehabilitation were to apply controlled stress to the repair site, to prevent adhesion formation, and to prevent potential gap formation in the repair site. Patients were instructed to perform controlled PIP joint motion with minimal active tension.

For the first 6 weeks following surgery, the patients with PIP joint transfixation (7 patients) and the patients with a static extension orthosis (3 patients) performed the DIP joint flexion exercises under the supervision of a hand therapist (C.A.K.). Active flexion exercises of the PIP joint to 30° were initiated immediately after the removal of the wire or orthosis at the end of the sixth week. If no extensor lag developed, the PIP joint flexion was progressed in 10° to 20° increments each week. Ten repetitions of each exercise were performed hourly. A volar static finger orthosis positioning the PIP joint at 0° was continued until 10 weeks after surgery. Active assistive PIP joint flexion exercises and combined wrist and finger flexion exercises were initiated at 8 weeks, and passive PIP joint exercises and strengthening exercises were started at 10 weeks. For some patients, light functional activities (<0.5–1.5 kg) could be started 8 weeks after surgery; however, forceful flexion and gripping activities were not allowed until 12 weeks after surgery.

For the remaining 3 patients, we used an RMF orthosis for 8 weeks. The RMF orthosis is designed to position the injured finger MCP joint in 15° to 20° more flexion than the adjacent fingers’ MCP joints. It permits MCP joint flexion but blocks MCP joint hyperextension. The concept is based on the fact that when an injured tendon is placed in 15° to 20° less relative motion than adjacent tendons from a shared muscle (extensor or flexor), there will be less force on the injured tendon than adjacent tendons. Therefore, placing the injured extensor hood digit in 15° to 20° more flexion than the adjacent fingers increases laxity in that profundus tendon, which relaxes that lumbrical from its downward pull while increasing tension on the extensor hood, encouraging dorsal repositioning.^10^ In addition, at night, patients wore a static finger orthosis that positioned the PIP joint at 0° at until the 10th week. The RMF orthosis positioned the MCP joint of the affected finger at 20° of flexion relative to the adjacent fingers. The aim of the RMF orthosis was to increase the activation of the extensor force on the PIP joint during PIP joint extension exercises and to prevent PIP joint extension lag.^10^ For the first 2 weeks, patients wore an RMF orthosis with a removable volar component that positioned the PIP joint at 0° of extension to prevent the development of extensor lag. Patients were advised to remove this component every 2 hours during the day to perform the PIP joint flexion exercises. An orthosis limiting PIP joint flexion to 30° was used during the PIP joint flexion exercises. If no extensor lag developed, the orthosis was progressed in average increments of 15° each week. The wrist was positioned at 30° of extension during the exercises. Proximal interphalangeal joint flexion-extension and DIP joint flexion exercises were performed every hour when the patient was awake. Composite flexion and gentle strengthening exercises were initiated between the fifth and sixth weeks.

### Outcome measurements

The outcomes were evaluated according to criteria for assessing standard functional results (active range of motion of the PIP and DIP joints; Table 2). The criteria for assessing standard functional results is based on active PIP joint flexion, PIP joint extension loss, and active DIP joint flexion measurements.^6^^,^^11^ The scores were defined as poor, fair, good, and excellent.^11^

**Table 2:** Evaluation of the Standard Functional Results

Loss of Active Extension (PIP Joint)		Active Flexion (DIP Joint)		Active Flexion (PIP Joint)		Results	
Degree	Score	Degree	Score	Degree	Score	Total Score	Functional Category
≤10°	0	≥70°	0	≥80°	0	0	Excellent
11°–20°	1	69°–40°	1	79°–60°	1	1	Good
21°–40°	2	<40°	2	<60°	2	2	Fair
>40°	3					3	Poor

## Results

Thirteen patients who met the inclusion criteria were included in the study: 7 men and 6 women, with an average age of 41 years (range, 20–71 years). Eight patients had an injured left hand and 5 had an injured right hand. All patients were right-handed. The deformity affected 8 index fingers, 4 middle fingers, and 1 little finger. The mean interval between the injury time and the surgery was 55.7 days (range, 15–180 days).

The mean follow-up period was 17.8 ± 10.27 months. The average PIP joint flexion was 104.8° ± 5.8° (range, 95° to 115°) and the loss of PIP joint extension was 6.15° ± 7.94° (range, 0°to 20°). The average DIP joint flexion was 65.38° ± 8.02° (range, 50° to 75°). Seven cases had excellent results, 4 patients had good results, and 2 patients had fair results (Table 1). No patients developed DIP joint hyperextension deformity. There were no complications. An example of a patient is shown in Video 1 (available online on the Journal’s website at www.jhandsurg.org) and Figure.

**Figure:**
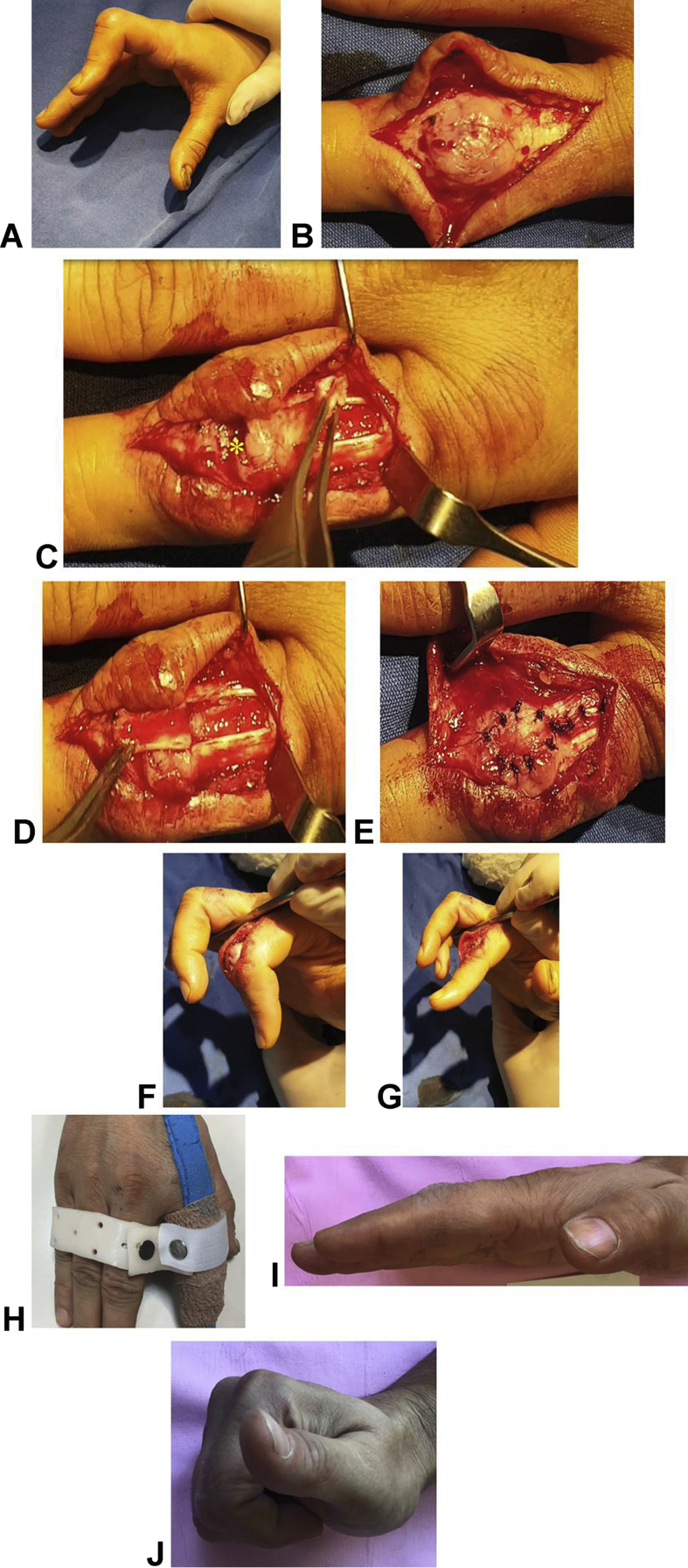
**A** Boutonniere deformity. **B** Fibrosis over the PIP joint. **C** Debridement of the fibrotic tissue (asterisk symbol) and elevation of the tendon flap to reconstruct the CS. **D** Insertion of the tendon flap to cover the PIP joint. **E** The flap and the defect of the extensor tendon were sutured. **F** Simulation of the RMF orthosis with a retractor. **G** Intraoperative active extension of the injured finger. **H** Patient’s rehabilitation with RMF orthosis. **I** Active PIP joint extension of the index (injured) finger at the third month after surgery. **J** Active PIP and DIP joint flexion of the index (injured) finger at the third month postoperatively.

## Discussion

Boutonniere deformity is among the most difficult extensor injury problems; this study demonstrated that surgical treatment of boutonniere deformity with a modified Snow technique under WALANT and a supervised rehabilitation period produced encouraging results in 11 (85%) of our patients.^10^ There are many studies for surgical treatment of CS injuries, and it is difficult to compare studies in which the patient population varies for each publication.

In previous studies, treatment of boutonniere deformity involved sacrificing another tendon. Harvesting a palmaris longus graft to reconstruct the severed CS resulted after surgery in 7° of PIP joint extension lag, but we achieved a similar extension lag without using a tendon autograft.^6^ Reconstruction of the CS with a flexor digitorum superficialis (FDS) tendon slip is a previously used method that resulted in 27° of PIP joint extension lag.^12^ Our technique resulted in an acceptable extension deficit without sacrificing any major tendon or making any tunnel in the phalanx. Palmaris longus tendon graft was used for the reconstruction of the CS in 11 cases, with 3 good results (27.7%).^3^ With our technique, we were able to achieve a high percentage of good and excellent results (85%).

The correction of the deformity by translocating the lateral bands dorsally and dividing the extensor expansion or mobilizing the transverse ligaments may be alternative choices, but both of these techniques fail to solve the main problem: a lack of CS in the injured finger.^13^^,^^14^ Although PIP joint extension lag was decreased to 7° by translocating the lateral bands dorsally, in our study, a similar extension lag was achieved without lengthening the scar to the DIP joint and without compromising the DIP joint extension.^13^ Mobilizing the transverse ligaments resulted in a notably higher PIP joint extension lag compared to our study (6.15° vs 21°, respectively).^14^ Probably, mobilization of the transverse ligaments is not effective enough to overcome the strong flexor forces at the PIP joint. Reconstruction of the CS with a structure close to the injury zone has the advantage that similar structural features exist in the donor zone, which can oppose strong flexor forces at the PIP joint. Also, primary repair of the donor zone over the proximal phalanx prevents the need to sacrifice any tendon. With all these surgical advantages and good results, we think that a modified Snow technique is a good choice for treating chronic boutonniere deformity.

Another important problem in boutonniere surgery is the limitation of the PIP joint flexion, probably because of the tight repair of the CS to overcome the extension lag. The average PIP joint flexion in our study was 104.8°. Central slip reconstruction with a palmaris longus autograft resulted postoperatively in 92° of PIP joint flexion.^6^ Mobilizing the lateral bands dorsally with different techniques resulted in 93.7° after surgery and 101° of PIP joint flexion.^11^^,^^14^ Central slip repair with an FDS tendon slip resulted in 80° after surgery of PIP joint flexion.^12^ We achieved more PIP joint flexion range of motion than other studies. We were able to check the stability and tightness of the PIP joint with active motion during surgery so that there would be no PIP joint flexion restriction due to the repair of the CS.

One of the aims of boutonniere surgery is to correct the DIP joint hyperextension deformity; in our study, none of the patients had postoperative hyperextension deformity of the DIP joint. In our technique, primary repair of the proximal donor area pulled the lateral bands dorsally via lateral slips of the extensor digitorum communis tendon, which helped correct the DIP joint hyperextension deformity. Also, the average DIP joint flexion in our study is 65.3°; this is a good range of motion for the DIP joint, allowing the patient to make a full fist and grab objects. Central slip reconstruction with a palmaris longus autograft resulted postoperatively in 55° of DIP joint flexion.^6^ Mobilizing the lateral bands dorsally with different techniques resulted in 75° after surgery and 38° of DIP joint flexion.^11,14.^Central slip repair with an FDS tendon slip resulted in 42.5° after surgery of DIP joint flexion.^12^ Flexion of the DIP joint is higher in our study than in other studies, except for 1 study in which extensor unit transection was done, which may enable more flexion but may cause DIP joint extension problems.^6^ The Snow technique is an effective method for correcting the boutonniere hyperextension deformity and results in better DIP joint flexion.

Besides the advantages to the surgical technique, the use of WALANT provided us with the ability to check the stability of the repair, predict the range of motion after surgery, and apply the pencil test, which enhances the rehabilitation protocol and provides intraoperative patient education. The pencil test is a simple test that simulates an RMF orthosis.^15^ A pencil was placed over the MCP joint of the affected finger to set the finger in a relatively flexed position. Then, the patient was requested to extend their finger. The test result was considered positive and an RMF orthosis was recommended if the PIP joint extension lag was improved with active extension of the finger. Regarding this philosophy, we also used RMF orthoses to decrease extension lag of PIP joints due to various finger injuries (Video 2; available online on the Journal’s website at www.jhandsurg.org). Supporting this, a recent prospective research reported hopeful outcomes.^16^ These advantages showed that WALANT is a suitable choice for boutonniere deformity correction surgery.

One of the limitations of the current study is the lack of a control group. The retrospective nature of the study is another weak point of our study. Finally, the heterogeneity of the postoperative orthosis fabrication protocol in our study is a limitation; this was because we were not aware of RMF orthoses in our initial cases. If possible, our preferred rehabilitation protocol is with RMF orthoses. Recently, Merritt and Jarrell^17^ reported excellent results in 23 patients treated with RMF orthoses for acute and chronic boutonniere deformities. We agree that RMF orthosis fabrication is a functional approach and affords hand use to increase range of motion.^17^^,^^18^

Our study demonstrated that a modified Snow technique is effective for CS injuries in which primary repair is not possible. Performing the surgery with WALANT enables the surgeon to check the stability of the repair to ensure a minimal PIP joint extension deficit so that early active motion with RMF orthoses can be started with confidence, and the surgeon can adjust the tightness of the repair so that maximum PIP joint flexion may be achieved.

## References

[1] Strauch R.J., Wolfe S.W. (2017). Green’s Operative Hand Surgery.

[2] Soldado F., De la Red-Gallego M.A., Barrera-Ochoa S. (2020). Dynamic transfer with the flexor digitorum superficialis for chronic boutonniere deformity reconstruction: a report of two cases. J Hand Surg Eur *Vol*.

[3] Suzuki K. (1973). Reconstruction of post-traumatic boutonniere deformity. Hand.

[4] Hou Z., Zhao L., Yu S., Xiao B., Zhou J. (2014). Successful surgical repair of central slip rupture in finger extensor tendon. *In* Vivo.

[5] Matev I. (1964). Transposition of the lateral slips of the aponeurosis in treatment of long-standing “boutonniere deformity” of the fingers. Br J Plast Surg.

[6] Duzgun S., Duran A., Keskin E., Yigit A.K., Buyukdogan H. (2017). Chronic boutonniere deformity: cross-lateral band technique using palmaris longus autograft. J Hand Surg Am.

[7] Snow J.W. (1973). Use of a retrograde tendon flap in repairing a severed extensor in the pip joint area. Plast Reconstr Surg.

[8] Williams K., Terrono A.L. (2011). Treatment of boutonniere finger deformity in rheumatoid arthritis. J Hand Surg Am.

[9] Ayhan E., Ozdemir E., Gumusoglu E., Cevik K., Eskandari M.M. (2018). The rise of wide awake hand surgery–contribution from Turkey. Hand Microsurg.

[10] Merritt W.H. (2014). Relative motion splint: active motion after extensor tendon injury and repair. J Hand Surg Am.

[11] El-Sallakh S., Aly T., Amin O., Hegazi M. (2012). Surgical management of chronic boutonniere deformity. Hand Surg.

[12] Patel S.S., Singh N., Clark C., Stone J., Nydick J. (2018). Reconstruction of traumatic central slip injuries: technique using a slip of flexor digitorum superficialis. Tech Hand Up Extrem Surg.

[13] Dubois E., Teboul F., Bihel T., Goubier J.N. (2017). Chronic boutonniere deformities, supple, or stiff: a new surgical technique with early mobilization in 11 cases. Tech Hand Up Extrem Surg.

[14] Ohshio I., Ogino T., Minami A., Kato H. (1990). Reconstruction of the central slip by the transverse retinacular ligament for boutonniere deformity. J Hand Surg Br.

[15] Lalonde D.H., Flewelling L.A. (2017). Solving hand/finger pain problems with the pencil test and relative motion splinting. Plast Reconstr Surg Glob Open.

[16] Wajon S, Howell JW. Prescription of exercise relative motion orthoses to improve limited proximal interphalangeal joint movement: a prospective, mulit-center, consecutive case series. *J Hand Ther*. Published online January 14, 2022. https://doi.org/10.1016/j.jht.2021.09.006. 10.1016/j.jht.2021.09.006 35039211

[17] Merritt W.H., Jarrell K. (2020). A paradigm shift in managing acute and chronic boutonniere deformity: anatomic rationale and early clinical results for the relative motion concept permitting immediate active motion and hand use. Ann Plast Surg.

[18] Lalonde D.H., Ayhan E., Gueffier X., Lalonde D.H. (2021). Wide Awake Hand Surgery and Therapy Tips.

